# Empirical study of daily link traffic volume forecasting based on a deep neural network

**DOI:** 10.1371/journal.pone.0327664

**Published:** 2025-07-03

**Authors:** Jin Ki Eom, Kwang-Sub Lee, Jin Hong Min, Ho-Chan Kwak

**Affiliations:** 1 Railroad Policy Research Department, Korea Railroad Research Institute, Uiwang-Si, Korea; 2 Department of Railway Management and Policy, Seoul National University of Science and Technology, Seoul, Korea; University of Shanghai for Science and Technology, CHINA

## Abstract

Forecasting the daily link traffic volume is critical in transportation demand analysis in feasibility studies for planning transportation facilities. The high purchase and maintenance cost of commercial transport planning software poses a challenge for several underdeveloped and developing countries. Therefore, there is a need for cost-effective methodology to forecast link traffic volume. This study proposes a data-driven approach for modeling traffic assignment and employs a deep neural network to forecast daily link volume derived from transport planning software. The main idea is that link traffic volume is significantly associated with traffic network attributes (i.e., number of lanes, travel speed, lane capacity, and roadway type) and network flow attributes (i.e., number of shortest paths on the corresponding link and origin-destination travel demand). Therefore, a multi-layer perception model is developed to effectively capture the nonlinear relationship among the link traffic volume, traffic network attributes, and network flow attributes. A case study demonstrated that the proposed method achieves comparable performance to commercial software in forecasting long-term link traffic volume. The obtained results indicated that the proposed method has the potential to serve as an alternative to commercialized software, although further studies are required to validate and enhance its application.

## Introduction

Currently, the application of artificial intelligence (AI) is expanding across all sectors and industries. There is a need to conduct basic research on the application of AI for forecasting link traffic volume for long-term transportation planning, in line with the current expansion of AI applications. In the transportation field, forecasting the link traffic volume of a transportation network is important from two aspects: long-term and short-term forecast, each serving distinct objectives [1]. Traffic forecasting is essential for establishing long-term urban transportation plans based on travel demand [2–5]. This strategic planning process involves forecasting long-term travel demand over periods exceeding 10 years, typically updated every 5 years. The predicted travel demand and traffic volume are crucial for conducting economic feasibility analysis of transportation projects. Therefore, the traffic volume is analyzed on a daily basis and segmented into peak/non-peak time periods. In addition, it is necessary to predict the traffic volume of all links in the network because the entire regional or metropolitan area network is the focus of the analysis. The traditional four-step demand estimation method is most frequently used to predict link traffic volume. This method culminates in the last of the four steps known as traffic assignment, where the estimated origin-destination (OD) demand for each zone pair is assigned to the traffic network. Although several formulations and techniques have been studied for solving the traffic assignment problem, the most fundamental approach is the user equilibrium (UE) method [3–7]. When travel demand and link traffic forecasts are analyzed for long-term planning, the main factors affecting them are demographic variables (increase or decrease in future population), land-use changes (development of new cities), and changes in transport infrastructure (opening new roads or transit routes).

In contrast, the short-term traffic volume prediction is frequently used for operational fields such as intelligent transportation systems (ITS) projects [8]. Because the main purpose is to smooth the flow of traffic and provide traffic congestion information in a timely manner, short-term forecasting with a shorter time horizon (e.g., 5–10 min) is important. Therefore, the day of the week, weather conditions, seasonal effects, and occurrence of large events are the major factors influencing short-term traffic volume prediction [1]. The main analysis target of short-term traffic volume prediction is not the entire city or regional network but is limited to small networks, such as one or multiple segments of corridors or highways [9]. Therefore, observed and historical traffic volume data obtained from GPS or loop detectors are important and are frequently used as input data for short-term traffic volume prediction. The prediction techniques used for this analysis include statistical methods [10–15] and deep learning models, which are a form of machine learning [1,16–22]. Recently, several studies have shown that predictive models using deep learning techniques are superior to existing statistical predictions [1,16,17,23–26].

However, the methodologies described above have some limitations. Because the dynamic traffic assignment (DTA) method incorporates a time-dependent OD matrix, it can present a more realistic traffic volume forecasting than the traditional UE-based travel model. However, it requires considerable input data, computation time, and cost owing to the higher computational complexity, making it impractical for large-scale, real-world network applications [26–29]. Mathematical analysis methods are inefficient for large-scale network analyses. Software packages such as EMME (Equilibre Multimodal Multimodal Equiibrium), TransCAD (Transportation Computer Assisted Design), and VISUM (Verkehr in Stadten-Umlegung) facilitate macroscopic analysis based on the traditional four-step travel demand forecasting method. However, owing to their limited flexibility, whenever there is a change in the OD matrix or transportation network, new or revised input data must be built, and the program must be run every time, which is cumbersome. In particular, it is necessary to budget for the costs of purchasing software and maintenance annually; therefore, it may be difficult for all agencies in underdeveloped and developing countries responsible for travel demand forecasting analysis to purchase the program. In contrast, earlier deep-learning-based methods have been widely used to predict short-term traffic volume for small networks because the model did not perform very well in terms of efficiency, accuracy, and computing costs when applied in a more complicated transport network [30]. Moreover, there is a notable absence of studies using deep-learning techniques and investigating their applicability in estimating large-scale link traffic for long-term transportation planning are lacking [1]. A contributing factor to the scarcity of such applications for large-scale networks is the impracticality of collecting the observed data for the entire network [31].

The study aims to establish a practical analysis methodology for estimating the link traffic volume of an entire city or regional network for long-term and macroscopic transportation planning, leveraging given travel demand (OD) estimates in the absence of observed traffic data. A significant challenge lies on how to develop a data-driven analysis framework that is efficiently applicable to large-scale real-world networks to explore the relationship between input data and link flows. Therefore, this study focuses on a methodology that uses a deep learning technique that has proven to be effective in short-term traffic volume forecasting. The main objectives of this study are to propose a novel link traffic forecasting methodology using a multilayer perceptron (MLP) model for large-scale networks and long-term traffic planning and to explore potential input data that can be applied conveniently and efficiently in practice rather than applying the theoretical approach of complex models. Specifically, we compared the prediction results using only network properties (i.e., number of lanes, roadway type, link speed, and lane capacity) as input data with those obtained by incorporating zonal OD and the k-shortest path algorithm to assess improvements in prediction accuracy and determine the optimal number of shortest paths in the model. Training data are required for model prediction; however, it is impossible to acquire observed data for the entire network in a city context. Therefore, we utilized the traffic assignment results obtained from the existing transport planning software, EMME, to study the traffic volume trends and empirically investigate the predictability of the proposed methodology. We used the transportation network of Uijeongbu City, Korea, as a case study.

The contributions of this study are summarized as follows:

(1) Present an empirical approach for large-scale macroscopic traffic forecasting by exploring the applicability of deep learning techniques, which have primarily been validated for short-term traffic forecasting, and proposing an analytical method that is more suitable for practical applications than complex theoretical models.
(2) Develop efficient and practical input data construction methods to enhance usability in large-scale networks, particularly by leveraging a network flow data construction method based on the k-shortest path algorithm. This approach enables continuous data preparation, independent of spatial and regional constraints, making it highly suitable for long-term traffic forecasting models.
(3) Improve forecasting accuracy by incorporating multiple factors, including traffic network attributes (i.e., number of lanes, travel speed, lane capacity, and roadway type) and network flow attributes (i.e., the number of shortest paths, and travel demand (OD matrices)).

This approach can be readily applied in underdeveloped and developing countries where observed traffic data are unavailable or access to transportation planning software is limited.

## Literature review

### Traffic volume prediction for strategic transportation planning

Traffic assignment methods, simulation-based models, and activity-based models are primarily used for analyzing long-term traffic demand and traffic volume forecasting for strategic transportation planning. Based on OD matrices, several different traffic assignment techniques are used in traditional four-step modeling, including all-or-nothing assignment [4], UE assignment [6], stochastic assignment [32], and DTA [27,33,34]. Rojo (2020) compared and evaluated different assignment methodologies using traffic data measured using detectors in a network in Barcelona, Spain. They observed that all-or-nothing and stochastic assignment methods were inadmissible in their particular case study, possibly owing to the large size of the network. The incremental assignment and successive averages algorithm were valid under certain conditions (i.e., more than 10 iterations). In contrast, the UE method was valid in all cases considered in their study.

Among the various techniques, DTA models captured time-dependent conditions in a network. Duell et al. [35] presented a large-scale DTA model for Sydney, Australia, with a model run time of approximately 48 h. Wang et al. [28] presented a comprehensive literature review of DTA for environmentally sustainable road transportation applications and discussed the future research prospects of DTA. Considering multiclass vehicles, public transit, and parking, Pi et al. [33] proposed a multimodal DTA model using the dynamic UE method and estimated the spatiotemporal passenger and vehicular flows. The experimental tests were conducted in the Pittsburgh region, USA. The proposed algorithm was tested using a large-scale network in Fresno, USA. However, the DTA model is a more complex algorithm requiring an iterative solution method for convergence. The performance of existing convergence algorithms in solving the DTA problem is sensitive to network size and saturation [27]. Studies have also estimated traffic volume using simulations [26] or activity-based modeling [36,37]. However, such models require a significant number of parameters and intensive input data, making it difficult to calibrate and validate the model in a large-scale network [26,33].

### Traffic volume prediction using statistics-based and deep learning-based models

Among the models used to estimate link traffic volumes, those based on statistical methods are relatively simple to use and easy to interpret. Typical statistics-based models include the smoothing method [12], autoregressive integrated moving average (ARIMA) model [11,38,39], and seasonal ARIMA (SARIMA) models [40,41]. However, they are not easy to adapt, are limited to simple cases, and require historically observed traffic flow data [16,24]. Kumar and Vanajakshi [10] developed a SARIMA model for the short-term prediction of traffic flow using only limited input data (i.e., the previous three days of traffic observations to predict the next day), whereas Luo et al. [42] proposed a hybrid method combining an improved SARIMA model with genetic algorithm optimization to predict short-term traffic flow.

Recently, several studies have been conducted using machine-learning and deep-learning techniques [23,30,43–45]. Boukerche et al. [24] compared two methods, statistics-based and machine-learning-based models, for short-term traffic flow predictions as an ITS application. They argued that statistics-based models have the advantage of model interpretability, whereas machine learning-based models are more flexible in terms of model adaptability. Polson and Sokolov [46] developed a deep-learning model for short-term traffic flow estimation. The model consists of two layers: the first uses a hierarchical sparse vector auto-regressive technique to identify spatiotemporal relations among predictions, and the other models nonlinear relations owing to transitions between free flow, breakdown, recovery, and congestion. Similarly, Wu et al. [47] proposed a DNN-based traffic flow prediction model to utilize weekly, daily, and spatiotemporal characteristics of traffic flow. Sun et al. [30] proposed a hybrid machine-learning-based model consisting of spatial pattern mining based on linear regression and a stacked gated recurrent unit to overcome the limitations of machine-learning-based models in complicated road networks. Mirzahossein et al. [48] proposed a short-term traffic volume estimation method based on a hybrid deep and machine learning model for three adjacent intersections and presented results showing that the gated recurrent unit (GRU) and long short-term memory (LSTM) bilayer network with wavelet transform (WL) noise reduction algorithm (WL + GRU-LSTM) achieved an accuracy of over 94%. More recent studies have proposed models to predict short-term traffic flows by considering individual travel frequencies [49] or incorporating weather factors such as temperature, rainfall, air quality index, and wind speed [50]. These studies have verified the performance of the proposed models through real-world case studies.

Machine-learning and deep-learning models have been reported to be effective tools for estimating traffic flow. However, most of them encountered few limitations: (1) limited use for a short term or on a small network because their main purpose was to use for ITS projects and not for transportation planning based on OD demand estimates; and (2) inevitably requiring a significant amount of training dataset, resulting in low efficiency, high computing costs, and concerns regarding the accuracy and reliability of the forecasting models [30,43,45]. Therefore, they are frequently used for short-term traffic flow prediction in small networks, such as single highways or ITS applications. Fafoutellis and Vlahogianni [43] indicated that the usability of these models is limited to small-scale networks because of data availability and quality, computational requirements, and model complexity. However, more recently, encouraging results have been reported. Rahman and Hasan [51] experimented with the traffic assignment problem on large-scale networks, such as Sioux Falls and East Massachusetts, using deep learning techniques. Given the OD demand and link flow, a neural network-based analysis technique known as the graph convolutional neural network (CNN) effectively learns UE traffic flows. Fan et al. [45] proposed a deep-learning-based DTA model for incomplete OD demand data. Testing the network in Chicago, U.S, revealed that the CNN-based DTA model exhibited higher accuracy than other statistical/machine learning algorithms (e.g., feed-forward neural network, k-nearest neighbor, and kriging).

## Methodology

### Analysis framework

**Fig 1:**
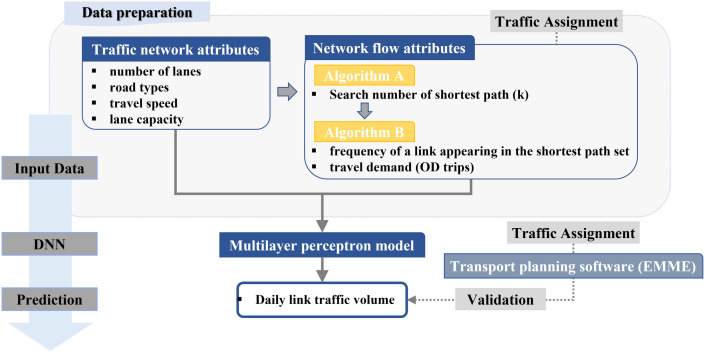
Research framework proposed in this study.

The research framework proposed in this study is illustrated in Fig 1. The data preparation process comprises two main components: (1) collection of the traffic network dataset and (2) execution of the k-shortest path algorithm to determine the frequency with which a given link appears in the shortest path set and to compute OD. The traffic network attributes (i.e., number of lanes, road types, travel speed, and lane capacity) and network flow attributes (i.e., number of shortest path and travel demand) are standardized to have a mean of zero and a standard deviation of one. The standardized input data, derived from the data preparation process, serve as input data for the MLP model, which is employed to forecast daily link traffic volume.

Unlike the network attribute data, obtaining observed traffic volume (y) for all links in the analyzed network was challenging. Traffic volume detection devices are not installed at every link, making it difficult to collect area-wide traffic volume data. Therefore, we utilized link traffic volume generated by existing commercial transport planning software (EMME). After executing the traffic assignment process in EMME using network attributes and OD demand as input data, the forecasted traffic volume from EMME was set as the target value and used to validate the performance of the proposed models.

### K-shortest path algorithm

The most important data added in this study are the shortest path data. Among the routes connecting individual departure points and destinations, the fastest route, that is the shortest route, was determined by considering the travel time (speed limit applied), and the number of times it was included in the shortest route was recorded for each link. To determine the most effective number of shortest paths for traffic forecasting, we considered the number of shortest paths from one to five. In general, links with a large number of shortest routes are expected to have high traffic volumes because most passengers prefer to take the shortest routes to reach their destination. Therefore, this study assumes that most travelers will use the shortest route connecting the departure and destination points and that alternative routes will be determined within the five shortest routes. Once each shortest path is determined, an OD demand is assigned to each link belonging to the shortest path as an input value.

At this time, more traffic is expected on the link belonging to the shortest path searched with the highest priority (e.g., k = 1) than on the link belonging to the shortest path searched with the lowest priority (e.g., k = 2 or more) (Table 1). Therefore, when generating count and OD, weights were introduced, and the amount was differentiated according to the order of each shortest path. For this purpose, the k-shortest path algorithm was used in this study because it is the most suitable algorithm for finding two or more shortest paths and their corresponding links. The weights applied in this study are as follows: in the case of finding only one shortest path (k = 1), the weight is 100%; for finding the two shortest paths (k = 2), the weights are 70% (the first shortest path) and 30% (the second shortest path); for finding the three shortest paths (k = 3), the weights are 50%, 30%, and 20%; for finding the four shortest paths (k = 4) the weights are 40%, 30%, 20%, and 10%; and for finding the five shortest paths (k = 5) the weights are 30%, 25%, 20%, 15%, and 10%, respectively. Whenever a link is included in the shortest path, the weighted count and OD are summed and stored in the individual link information.

**Table 1:** K-shortest path algorithm, finding count, and OD algorithm.

**Algorithm A**: Search k-shortest path
input: Link information and O/D in Uijeongbu City output: Shortest path by O/D 1. set O,D; 2. function find_multi_shortpath() 3. Read Link_Info,O,D; 4. for (i = 0;i < O;i++) 5. for (j = 0;j < D;j++) 6. Find ShortestPath(Link_Info,O,D,k); 7. save result (O,D,Path_ID,Link_ID,Path_Order,cost,agg_cost); 8. end function;
**Algorithm B**: Finding count & OD for each link when k = 5
1. function calculate_link_OD_5() 2. calculate link Sum_link_ount_OD(Link_ID,sum(OD) as OD,count) case path_id = 1 then final_count = count * 0.3 final_OD = OD * 0.3 case path_id = 2 final_count = count * 0.25 final_OD = od * 0.25 case path_id = 3 final_count = count * 0.2 final_OD = OD * 0.2 case path_id = 4 final_count = count * 0.15 final_OD = OD * 0.15 case path_id = 5 final_count = count * 0.1 final_OD = OD * 0.1 3. end function

**Fig 2:**
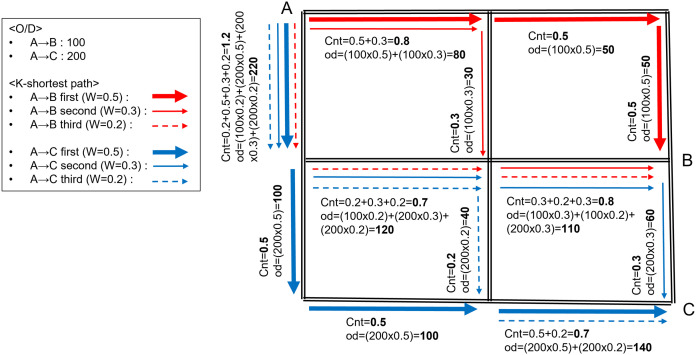
Calculation example of short path counts and od trips with k  =  3.

A simple example of this process is shown in Fig 2, illustrating how count and OD for each link is calculated according to the weight of each shortest path to determine the three shortest paths (k = 3) when the OD from zone A to zones B and C are 100 and 200, respectively.

### Model structure and deep learning process

We compared the results according to the configuration of network data for practical use in the field, which significantly influenced traffic assignment, i.e., traffic volume forecasting. Therefore, as the basis of this study, a general MLP was introduced to compare the suitability of traffic volume prediction according to network data, travel demand (OD), and the number of shortest paths, which are critical factors for traffic assignment in the traditional approach.

MLPs are widely employed to explore potential relationships between input variables (X) and target values (Y) [52]. They enable computational models to be structured into multiple processing layers, facilitating the learning of data representations at various levels of abstraction [53].

Although models such as LSTM and GRU are suitable for time-series prediction tasks, they were not applicable in this study because the dataset does not contain temporal sequences. Each observation represents the traffic volume on a given link between node pairs (i, j), without any sequential dependency. Furthermore, due to the spatial sparsity of links and nodes in the large-scale network, convolution-based architectures (e.g., CNNs) were also deemed unsuitable. In this context, MLPs were chosen for their effectiveness in modeling nonlinear relationships in proposed dataset.

**Fig 3:**
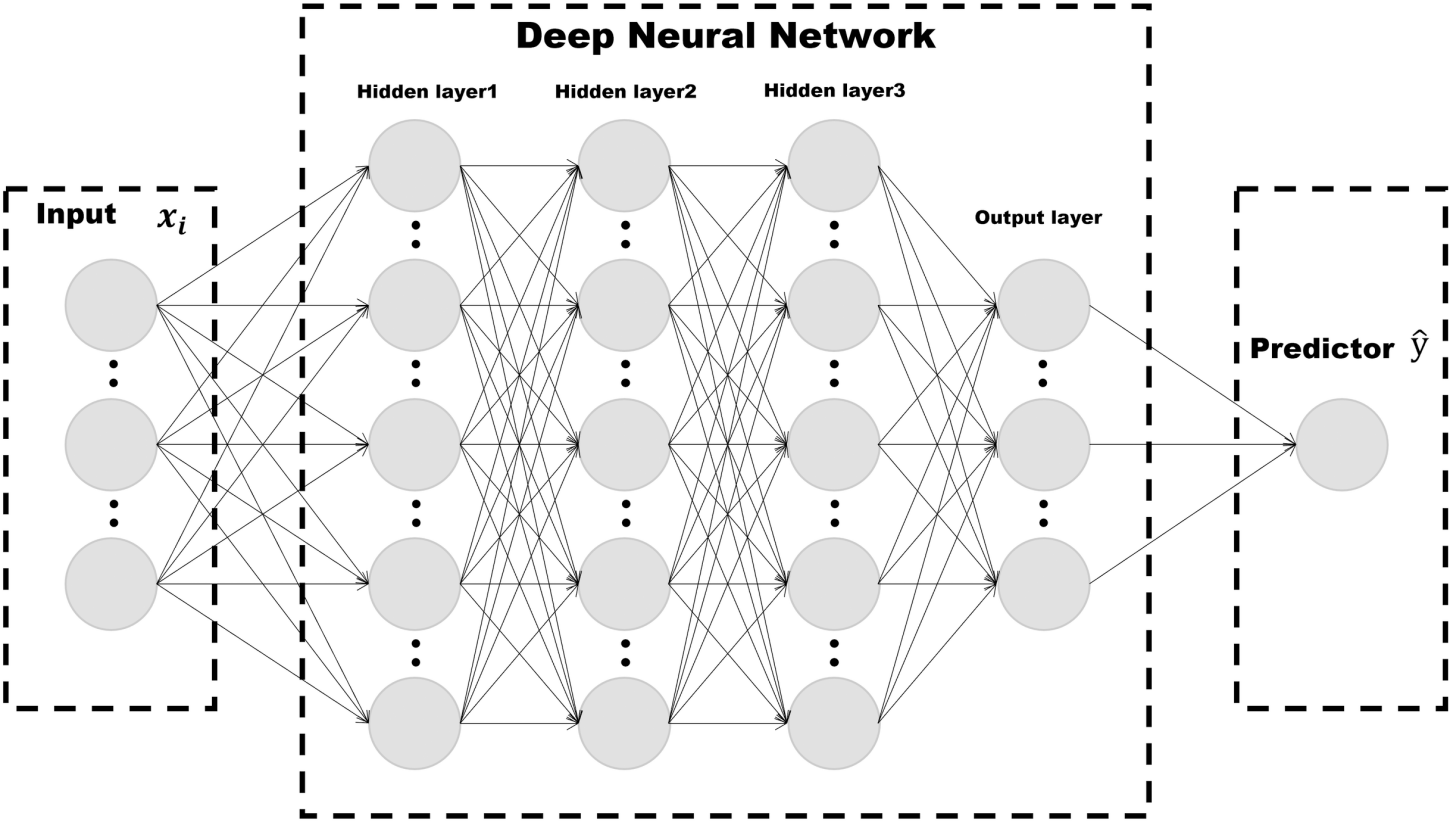
Concept of MLP network.


Given these properties, we implemented an MLP, which is commonly utilized in regression analysis. The MLP structure comprised an input layer, hidden layers, an output layer, and various activation functions [54]. Each neuron in the input and hidden layers is connected to all neurons in the next layer, as shown in Fig 3. The basic function of an MLP in predicting the link traffic volume is expressed as follows:

$$y=f\left(x\cdot W_{1}+b_{1}\right)$$ (1)

where, f denotes the activation function, $W_{1}$ is the weight matrix, and $b_{1}$ is the bias vector.

We used the batch training approach and the rectified linear unit (ReLU) function as the activation function of the hidden layer for this analysis. The traffic volume on any given link can be obtained from the output node after inputting a combination of contextual factors into the predictor for a particular link. Because of the inconvenience of the prediction process, as only one forecasted traffic volume is obtained at a time, we use the same matrix operation as the batch training. A factor matrix ($x_{i}$) is added to the model, and a predicted traffic volume matrix ($\hat{y}$) can subsequently be output at the final layer. As expressed in Equation (2), each matrix ($x_{i}$) consists of several factors ($x_{ij}$), which are the primary factors influencing traffic volume estimation. The batched matrix format of the MLP model is expressed as follows:


$$\left[\begin{array}{c} {\hat{y}}_{1}\\ \vdots \\ {\hat{y}}_{n} \end{array}\right]=[\begin{array}{ccc} x_{11} & \cdots & x_{1n}\\ \vdots & \ddots & \vdots \\ x_{m1} & \cdots & x_{mn} \end{array}]\cdot \left[\begin{array}{c} w_{1}\\ \vdots \\ w_{n} \end{array}\right]+\left[\begin{array}{c} b_{1}\\ \vdots \\ b_{n} \end{array}\right]$$ (2)

where n denotes the number of batch sizes and m is the feature number of each input sample.

After training the model, we used the finalized parameters of the MLP to forecast the link traffic volume for the validation data and evaluated the performance of the predictive capability based on the network and flow properties.

After standardizing the data by subtracting the mean and dividing by the standard deviation, the dataset was divided into three subsets: training (64%), validation (16%), and testing (20%). To optimize predictive performance, hyperparameter tuning was conducted through empirical testing. This involved adjusting the number of neurons in each hidden layer, the learning rate, batch size, and other model-specific parameters. Table 2 summarizes the architecture and training configuration of the proposed MLP model, including the layer architecture, activation functions, loss function, and optimization strategy. The model consists of two hidden layers with ReLU activation, and is optimized using RMSprop with a learning rate of 0.001. Training was conducted with a batch size of 32, using mean absolute error (MAE) as the loss function. Early stopping was applied based on validation loss to prevent overfitting. Model development was conducted using Python 3.8, machine learning platform TensorFlow, and programming interface (API) Keras.

**Table 2:** Hyperparameter configuration of the proposed neural network model.

Component	Configuration/value
Input layer	number of features = 9
Hidden layers	Dense (64 units), Activation: ReLU
Output layer	Dense (1 unit), Activation: Linear
Optimizer	RMSprop, Learning Rate = 0.001
Loss function	Mean Absolute Error (MAE)
Batch size	32
Epochs	1000
Validation split	20% (of training data)
Early stopping	Applied on validation loss, Patience = 10 epochs

## Case study

### Study area

The experimental site of this study was Uijeongbu. It is located in the northeastern part of Gyeonggi and serves as the administrative center of northern Gyeonggi, hosting offices of the Gyeonggi provincial government and education departments. The city borders Seoul, the capital city of Korea, to the south and has an area of approximately 81.54 km^2^. Since the 1990s, large-scale apartment complexes have been developed in the eastern part of Uijeongbu, and the population is gradually increasing, with approximately 460,000 residents in 2021. There are three major railway lines (e.g., Metropolitan Subway Line 1, Seoul Subway Line 7, and Uijeongbu Light Railroad), city and intercity buses are in operation, and two highways and three express roads form the main roads. To minimize the impact of external traffic, a case study was conducted focusing only on the internal road network, excluding two highways and three express roads. According to the National Transport DB [55], the Uijeongbu City network comprises 15 zones, including 1,432 nodes and 3,179 links.

### Data preparation and description

In this study, the most important and basic input variables for traffic volume prediction were traffic network data. Network data provide the identity of each individual link connecting standard nodes, and link-specific properties, including the number of lanes, road types, travel speed, and link capacity, provided by KTDB, 2022. The link traffic volume is affected by the OD trips represented by the traffic demand, and this OD demand is assigned to the network by the shortest path (considering link travel time) connecting the origin and destination. The basic idea behind using network flow attributes is that the OD between zones has a close relationship with the link traffic volume, and that travelers follow the shortest path to reach their destination the fastest. Therefore, it is necessary to prepare data on the shortest route between each departure node and destination, and the OD demand that can be used on that route. Multiple shortest paths between individual departure nodes and destinations can be designated and it is necessary to prepare data for the OD ratio according to the number of shortest paths. This method of constructing network flow data has the advantage of being able to prepare data at all times, regardless of the region and space, when developing a model for long-term traffic forecasting.

Table 3 lists an example of the input data (case of shortest path k = 3) for forecasting traffic volume using the MLP model. The mean and maximum values of the link traffic volume as a target variable were 710 vehicles and 4,144 vehicles per day, respectively. The number of lanes varied from 1 to 5 per direction. The travel speed ranged between 20 and 100 km/h, and the average speed was 45.2 km/h. Lane capacities ranged between 595 vehicles/h/lane and 2,028 vehicles/h/lane, with an average capacity of 1,014 vehicle/per hour/per lane. Based on the shortest path algorithm used on the network data, we observed that the average shortest path count per link was 3.4 with a maximum of 11.0. At the shortest path k = 3, the traffic demand (of trips) ranged from 0 to 3,254 trips. The shortest path count per link depends on the number of routes, and the number of trips in each link varies depending on the counts of the shortest paths calculated.

**Table 3:** Finalized input data description used in MLP application with k = 3.

Variable	Description	Mean	Max.	Min.	Unit
Y(volume)	Link volume (traffic volume by each link)	710.0	4,144.0	1.0	vehicle/day
X(lane)	No. lanes (number of lanes per direction)	2.1	5.0	1.0	lane/direction
X(speed)	Link speed	45.2	98.3	27.7	km/h
X(capacity)	Lane capacity (maximum number of vehicles passing through per hour per lane)	1,014.0	2,028.0	595.0	vehicle/h/lane
X(count)	No. counted shortest path (Number of times a specific link is included in the shortest path)	3.4	11.0	0.0	frequency
X(OD)	Travel demand	856	3,254	0.0	trips

Table 4 summarizes the correlation matrix between the input variables and the output value (i.e., traffic volume). Among the input variables, the count and OD vary based on the number of shortest paths (k). This study considers one to five shortest paths between OD pairs. It is widely accepted that the correlation value exceeding 0.7 indicates a significantly strong correlation, while a value in the range of 0.4–0.7 reflects a moderate to strong correlation. The positives (+) and negative (-) signs denote whether the correlation is positive or negative, respectively. As expected, the table presents that the number of lanes, travel speed, and lane capacity correlate more strongly to the traffic volume (0.48–0.63) than other input variables while the count, and OD exhibit relatively low correlation to the traffic volume (0.2–0.3). These results are expected because the number of lanes, travel speed, and lane capacity can be considered as factors that directly affect traffic volume. In contrast, the number of shortest paths and OD can be defined as factors that have an indirect effect. In terms of the correlation according to the number of shortest routes, the correlation between traffic volume variables and individual variables was observed to be highest on average when the number of shortest routes was three (k = 3). As the number of shortest routes (count) increases to four or more, the correlation between the travel speed and traffic volume appears to decrease, whereas the correlation with count increases.

**Table 4:** Correlation matrix for output data (traffic volume) and input variables with k.

k = 1	Lane	Speed	Capacity	Count	OD	Volume
Lane	1					
Speed	0.5785	1				
Capacity	0.6016	0.7698	1			
Count	0.1193	−0.0010	0.0628	1		
OD	0.2002	0.0846	−0.0254	0.8636	1	
Volume	0.5506	0.1793	0.5821	0.2999	0.2430	1
k = 2						
Lane	1					
Speed	0.6602	1				
Capacity	0.5688	0.9707	1			
Count	0.1464	0.0670	0.0662	1		
OD	0.1921	0.0263	−0.0006	0.8879	1	
Volume	0.4891	0.5385	0.5464	0.2858	0.3002	1
k = 3						
Lane	1					
Speed	0.7189	1				
Capacity	0.6428	0.9723	1			
Count	0.2070	0.0955	0.0859	1		
OD	0.2244	0.0530	0.0257	0.9046	1	
Volume	0.6078	0.6206	0.6247	0.2174	0.2078	1
k = 4						
Lane	1					
Speed	0.6662	1				
Capacity	0.5752	0.9709	1			
Count	0.1734	0.0655	0.0663	1		
OD	0.2148	0.0306	0.0071	0.8984	1	
Volume	0.4640	0.5567	0.5672	0.2559	0.2726	1
k = 5						
Lane	1					
Speed	0.5474	1				
Capacity	0.6032	0.7179	1			
Count	0.2164	0.1398	0.1546	1		
OD	0.2415	0.1637	0.1103	0.9308	1	
Volume	0.5511	0.1700	0.5803	0.2812	0.2578	1

## Results and validation

### Prediction performance evaluation

**Fig 4:**
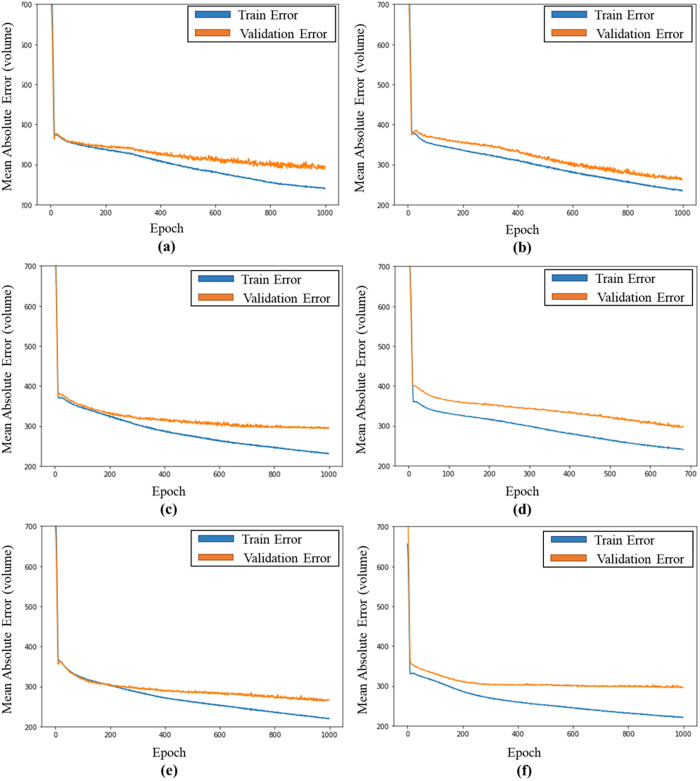
The trend of MAE with validation and testing data. (a) case 0:no short path, (b) case 1: k = 1, (c) case 2: k = 2, (d) case 3: k = 3, (e) case 4: k = 4, (f) case 5: k = 5.

To evaluate the stability of the models, we monitored the variation in training and validation loss values over 1,000 iterations. Fig 4 presents the learning curves for each case based on the number of shortest paths (k). We observed that it took approximately 200 iterations for the model to converge to a stable solution, after which there were no variations in the loss values. If the difference in MAE values did not decrease as the number of iterations increased, we used the EarlyStopping callback option with the root mean square propagation optimizer.

As shown in Fig 4(a), when only the traffic network data were used, the validation error did not decrease after converging at approximately 20 iterations. However, the models applying the number of shortest paths and OD values indicated that the validation error continued to decrease (Figs 4(b),4(f)). In particular, when the number of shortest paths was three (k = 3), the validation and training errors decreased as the number of learning repetitions increased. In contrast, when the number of shortest paths was five (k = 5), the number of learning repetitions decreased. It can be observed that the validation error no longer decreases even as the number increases. Moreover, the gap between the training and validation errors increases.

These results indicate that simply increasing the number of shortest routes cannot be considered advantageous for predicting traffic volume. Increasing the number of shortest paths means that the time and cost to calculate the OD value and include it in the link attribute increases with the number of shortest paths. Therefore, it was observed to be meaningful in terms of increasing the efficiency of data construction by appropriately including the number of shortest routes and simultaneously improving the accuracy of traffic volume prediction.

### Evaluation metric

To evaluate the performance of the traffic volume forecasting according to the number of shortest paths between OD, this study uses the most common three performance measures, i.e., $R^{2}$, root mean square error (RMSE), and MAE. $R^{2}$ considers the proportion of traffic volume variance from the prediction models and provides a value to measure how well the model can replicate the real traffic volume. To obtain the coefficient of determination ($R^{2}$), the residual sum of squares (RSS) and total sum of squares (TSS) are calculated as follows:


$$\text{RSS}=\sum_{i=1}^{n}{\left(y_{i}-{\hat{y}}_{i}\right)}^{2}\mathrm{\backslash ]}$$ (3)

$$\text{TSS}=\sum_{i=1}^{n}{\left(y_{i}-\overset{―}{y}\right)}^{2}\mathrm{\backslash ]}$$ (4)

$$R^{2}=1-\frac{\text{RSS}}{\text{TSS}}$$ (5)

where $y_{i},\hat{y_{i}},$ and $\overset{―}{y}$ denote the true value, predicted value, and mean of the true value, respectively.

RMSE is a measure of the average magnitude of the errors between the predicted and actual values, giving higher weight to larger errors. It is defined mathematically as the square root of the average squared difference between predicted and actual values. The RMSE for n predictions are calculated as follows:


$$\text{RMSE}=\sqrt{\frac{1}{n}\sum_{i=1}^{n}{\left(y_{i}-{\hat{y}}_{i}\right)}^{2}}\mathrm{\backslash ]}$$ (6)

where $y_{i}$ denote the true value of the traffic flow of link $i,{\hat{y}}_{i}$ is the predicted value of the traffic flow of link i.

MAE, in contrast, measures the average absolute error between the predicted and actual values. Unlike RMSE, MAE treats all errors equally, providing a straightforward measure of prediction accuracy. It is expressed as follows:


$$\text{MAE}=\frac{1}{n}\sum_{i=1}^{n}\left|y_{i}-{\hat{y}}_{i}\right|\mathrm{\backslash ]}$$ (7)

where $y_{i}$ denote the true value of the traffic flow of link $i,{\hat{y}}_{i}$ is the predicted value of the traffic flow of link i.

The RMSE is sensitive to outliers as it tends to penalize larger errors more severely, whereas the MAE provides a simple average error magnitude. Both RMSE and MAE provide a comprehensive assessment of the prediction performance of the model, with RMSE emphasizing the accuracy of the model across the entire range of data and MAE providing a measure of the central tendency in the prediction errors. $R^{2}$ ranges between 0 and 1, with higher values indicating better model performance. In contrast, MAE and RMSE exhibit an inverse relationship with model performance, where lower values correspond to better accuracy. These metrics were calculated for each model across all the scenarios to facilitate a robust comparison of their predictive capabilities.

**Fig 5:**
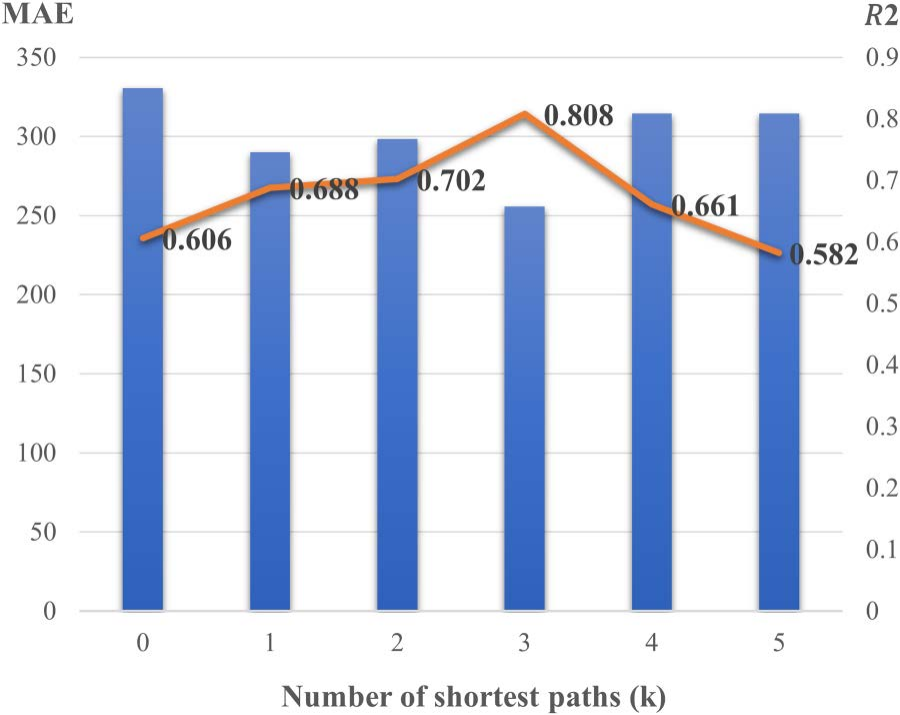
The performance results of each model based on MAE and $R^{2}$.

This study conducted two experiments and developed six models. In the first experiment, link traffic volume was predicted through the MLP model using only basic traffic network properties, whereas two additional variables were generated and used for prediction in addition to the network flow variables in the second experiment. The process of generating the two-variable data is as follows: After determining the k-shortest path between zones and considering whether the link belongs to the shortest path for each OD, the number of times the link belongs to the shortest path (variable name: count) and the OD demand between zones (variable name: OD) were generated as input variables. Table 5 and Fig 5 summarize the results of the cases reflecting the number of shortest path implementations with the performance metrics, including RMSE, MAE, and $R^{2}$. All three performance indices exhibited the best accuracy for the three shortest paths (k = 3) with respect to the highest $R^{2}$ (0.808), lowest MAE (255.6 (vehicle/day)), and RMSE (370.0 (vehicle/day)).

**Table 5:** Summary of model performances based on the number of shortest paths.

Descriptions	Case	MAE (vehicle/day)	RMSE (vehicle/day)	$R^{2}$
The number of shortest paths (k)	0	330.6	453.5	0.606
	1	289.9	420.1	0.688
	2	298.4	426.9	0.702
	3	255.6	370.0	0.808
	4	314.4	448.3	0.661
	5	314.3	468.3	0.582

As shown in Fig 5, the MAE is largest when there is no shortest path (k = 0), and this value decreases until there are a particular number of shortest paths (k = 4). $R^{2}$ was 0.606 at k = 0 and it increased to 0.808 at k = 3, which was the highest value of $R^{2}$. Afterwards, as the number of shortest paths increased, the MAE increased and $R^{2}$ decreased.

These findings have several important implications. As presented in the previous section, when preparing data for traffic volume prediction, it is necessary to divide the OD demand according to the number of shortest paths. This is because, as the number of shortest paths increases, the time and cost required to divide the OD demand increases.

In the entire network, the shortest path between the departure points and all destination points must be determined, and a specified number of times is stored in each link, which is designated as the shortest path. The OD demand value for each link is divided according to the number of stored shortest paths, and because most OD demand values are assigned to links designated with a high frequency, the computer computation time increases as the number of shortest paths increases. Moreover, in large cities, the number of links included in traffic volume prediction is significantly high; therefore, it is believed that there will be limitations in applying the method proposed in this study for practical use. The results of this study show that adding an input variable for the OD to the link included in the shortest path increases the prediction accuracy and that the prediction accuracy does not necessarily increase because the number of shortest paths (k) is large. Therefore, it is essential to determine the number of efficient shortest routes needed to predict the traffic volume.

### Model performance comparisons based on visualization

**Fig 6:**
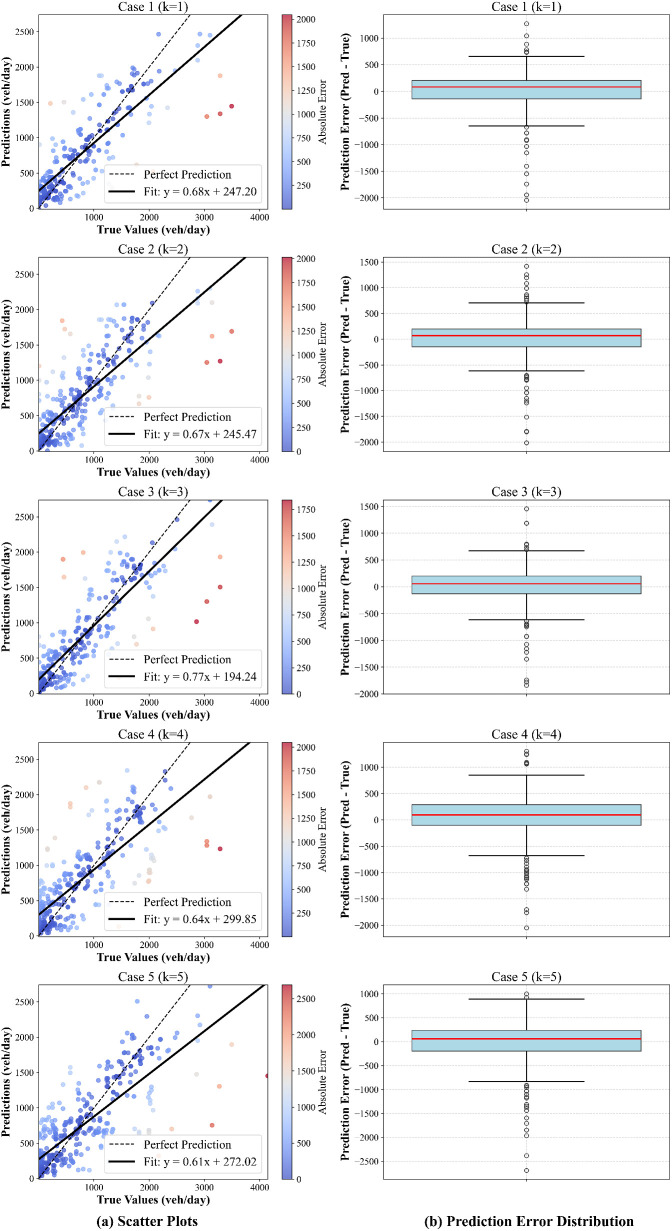
Model prediction performance for different cases (k =  1–5).

Fig 6 presents the prediction performance of five different models (k = 1–5), where each row corresponds to a different case. The left column (a) illustrates the scatter plots comparing the true values (vehicle/day) and the predicted values, while the right column (b) displays the box plots representing the distribution of prediction errors (i.e., |Prediction−True|). The scatter plots illustrated in Fig 6(a) provide a visual comparison between actual and predicted values. The black dashed line represents the perfect prediction line (y  = x), indicating the ideal scenario where all predictions perfectly match the true values. The solid black line corresponds to the linear regression fit of the predictions, reflecting potential biases and deviations from the ideal case. A notable deviation from the perfect prediction line suggests systematic prediction errors. Furthermore, the color gradient of the data points represents the absolute error, with blue indicating lower errors and red representing larger discrepancies. This visualization allows for an intuitive assessment of model accuracy across different ranges of vehicular flow.

The box plots illustrated in Fig 6(b) provide an overview of the prediction error distribution for each model. The red line within each box represents the median error, while the interquartile range (IQR) denotes the variability in prediction accuracy. The whiskers extend to 1.5 times the IQR, and data points outside this range are considered outliers, indicating instances of significantly large prediction errors. Models exhibiting a narrower IQR and a median closer to zero suggest improved predictive performance and reduced bias. However, the presence of numerous outliers in certain cases highlights instances where the model struggles to generalize effectively.

A comparative analysis across different cases reveals that the model with k = 3 demonstrates the best overall performance in terms of both bias and variance. For k = 3, the model achieves the best balance between prediction accuracy and stability. The regression fit line closely aligns with the perfect prediction line, and the corresponding error distribution is more compact with fewer extreme outliers. These findings highlight the importance of selecting an optimal model complexity to enhance predictive performance. Further studies could explore hyperparameter tuning, ensemble learning techniques, or feature selection strategies to refine the model and reduce extreme prediction errors.

**Fig 7:**
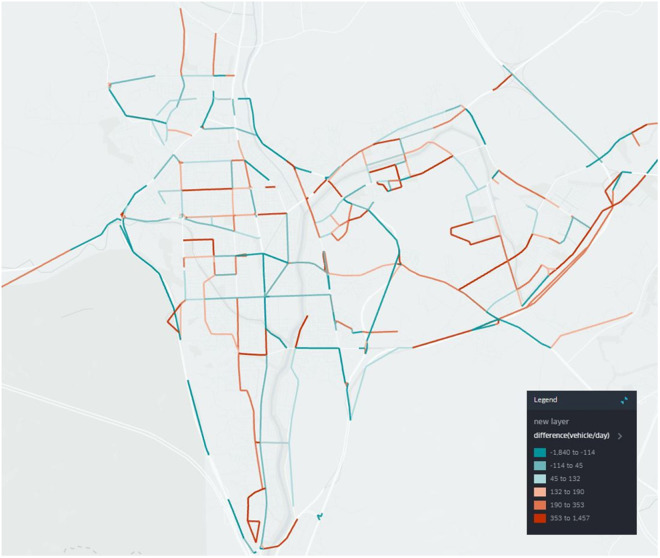
Difference between prediction and true traffic volume (k = 3, vehicle/day) using Kepler.gl (https://kepler.gl/).

Using an open-source geospatial analysis tool, Kepler.gl (https://kepler.gl/), we plotted the roadways with the difference between true traffic volume and predicted one (where the prediction errors is lowest case, k = 3) in Fig 7. The difference between the predicted traffic volume and the reference traffic volume in some roadway sections was shown as a positive or negative value. It was difficult to identify characteristics of the difference between the predicted and true values by roadway sections. However, overall, we observed that the finest model predicted the arterial roadways sections to have relatively low traffic volumes, whereas the local roadways within the district level predicted to have high traffic volumes. Based on these results, it is essential to explore ways to enhance the accuracy of traffic volume prediction according to roadway levels in the future. For example, one approach would be to evaluate data that is easily obtained from the field such that the difference in traffic volume between roadway functions can be reflected. In addition, it is necessary to reflect such differences in roadway levels through application of alternative deep learning approaches.

## Conclusions

In this study, as part of the basic research, we proposed a novel link traffic forecasting methodology using a deep learning technique for large-scale networks and long-term traffic planning. We explored potential input data that can be conveniently and efficiently applied in practice rather than applying a theoretical approach to complex models. We tested a new prediction method for daily long-term traffic volume based on determining the relationship between the number of shortest paths and traffic volume using MLP network.

We presented an algorithm for data construction on the shortest route between each departure point and destination, and the OD demand that can be used on that route. Multiple shortest paths between individual departure points and destinations were designated, and the OD demand was determined according to the number of shortest paths (from one to five). In addition, to overcome the limitation of the acquisition of the observed traffic volume (y) for all links in the analyzed city, we utilized link traffic volume generated by existing commercial transport planning software (EMME). After executing the traffic assignment process in EMME using the network attributes and OD demand as input data, the forecasted traffic volume from EMME was set as the target value.

This method of constructing data has the advantage of being able to prepare data at all times, regardless of region or space, when developing a model for long-term traffic forecasting. After data preparation, we applied the MLP model to predict traffic volume on each link, reflecting the number of shortest path (k = 0–5) implementations with the performance measures, including RMSE, MAE, and $R^{2}$. All three performance measures exhibited the best accuracy for the three shortest paths (k = 3) with respect to the highest $R^{2}$ (0.808), lowest MAE (255.6 vehicle/day), and RMSE (370.0 vehicle/day). However, when the number of shortest paths was more than four, the size and frequency of errors increased. The visualization results indicated that the model with k = 3 provides the best trade-off between bias and variance, achieving both accuracy and stability. Models with lower k values exhibit underfitting, while models with higher k values introduce increased variance, resulting in larger extreme errors. This indicates the importance of determining an appropriate number of shortest paths required for traffic volume prediction. Further increasing the number of shortest paths can result in a significant increase in the computer computation time.

In future research, the significant variations in traffic volume for roads with similar properties across different city sizes need to be addressed. This highlights the necessity of developing traffic volume prediction models that can be generalized across various urban environments. To achieve this, it is essential to explore methodologies that can effectively capture spatial and structural variations in road networks across various cities. Furthermore, to enhance model performance and adaptability, future studies should explore alternative deep learning approaches. One promising direction is the application of graph neural networks (GNNs), which leverage graph theory to model complex relationships within a network. Unlike conventional deep learning models, GNNs can effectively represent the topological properties of a road network by capturing interactions between nodes (e.g., intersections) and links (e.g., road segments). By incorporating the connectivity and spatial dependencies of road networks, GNN-based models have the potential to improve the accuracy and robustness of traffic volume predictions, particularly in diverse urban settings. By integrating such advanced techniques, future research can contribute to the development of more scalable and transferable traffic prediction models, ensuring their applicability to cities of different sizes and structures.

## Supporting information

S1 Data Input dataset. (XLSX)
